# Determining female‐specific high‐intensity activity GPS thresholds in women's rugby union: Use of current use of male‐derived absolute speed thresholds underestimates true levels

**DOI:** 10.1002/ejsc.12149

**Published:** 2024-06-17

**Authors:** Eddie Bradley, Jenny Roberts, David Archer

**Affiliations:** ^1^ University of Sunderland Sunderland UK

**Keywords:** female sport, locomotion, match demands, maximum speed

## Abstract

GPS speed thresholds in women's rugby union are usually based on data derived from the men's game. However, evidence suggests the maximum speeds achieved by female players are 2–8 km.h^−1^ slower and the volume of high‐intensity running (HIR) in women's rugby may be underestimated. The aim of the study was to examine the effect of adjusting absolute thresholds on the volume of high‐intensity locomotion. GPS units recorded movement data from 58 players across 18 English Premier15 s matches. Distance in HIR and sprint (Spr) zones were calculated using male‐derived criteria: Abs_Male_ (HIR >18 km.h^−1^; Spr ≥21 km.h^−1^). Two alternative thresholds were compared: Abs_FVmax_ (HIR >16 km.h^−1^; Spr ≥19 km.h^−1^); Abs_Female_ (HIR >14 km.h^−1^; Spr ≥17 km.h^−1^). Data were analysed using one‐way ANOVA and effect sizes to determine differences in distances between thresholds. Abs_Male_ HIR and Spr distances were 63 ± 71 m and 30 ± 53 m. Significantly greater distances at higher‐intensity speeds were observed with female‐adjusted thresholds. Abs_FVmax_: HIR: 139 ± 116 m (*p* = 0.01, ES 0.80); Spr: 60 ± 90 m (*p* = 0.131, ES 0.41) and Abs_Female_: HIR: 239 ± 157 m (*p* < 0.01, ES 1.45); Spr: 137 ± 152 m (*p* < 0.01, ES 0.94). 24 players (41%) achieved speeds greater than the 21 km·h^−1^ threshold with the male‐derived thresholds. At Abs_FVmax_ threshold this increased to 44 (76%) and 100% at the Abs_Female_ threshold. Existing male‐derived thresholds appear to underestimate high‐intensity locomotion of female players. With adjusted thresholds, specifically the Abs_Female_, the proportional volume of high‐intensity activity in the women's game (8.2% total distance) aligns more closely to that observed during men's match‐play.

## Highlights

- Calculation of the total high‐intensity locomotion in women's rugby union (RU) using male‐derived thresholds (92.3 ± 123.8 m) equates to 2.1% of the total distance covered in games.
- Adjusting thresholds relative to the speed at the second ventilatory threshold and female maximum velocity increased the proportion of high‐intensity activity to 4.5% and 8.2% of total distance respectively.
- Reducing GPS high intensity speed thresholds to >14 km.h^−1^ for high‐intensity running (HIR) and ≥17 km.h^−1^ for sprinting produce high‐intensity speed distances that are proportionally similar to male rugby players and are more suitable when analysing match demands in women's RU.

## 1 INTRODUCTION

Rugby union (RU) is classified as an intermittent field‐based sport that requires a high level of aerobic and anaerobic demand, combined with periods of intense effort <2 min in length (Sheppy et al., 2019), with work to rest ratios reported as high as 1:5.7 and 1:0.7 in men's and women's RU respectively (Cunniffe et al., 2009; Suarez‐Arrones et al., 2014) with the high variability in these measures explained by the differences in ‘work’ thresholds used. High‐intensity play has been reported as a key determinant for success in rugby (Bremner et al., 2013; Hughes et al., 2017), for example, more frequent repeated high intensity efforts (Gabbett, 2013), collisions (Nolan et al., 2023; Woodhouse et al., 2022) and faster ruck speed (Bremner et al., 2013) are observed in successful teams. Increased running speed is likely to result in a greater number of line breaks (Smart et al., 2014), alongside creating more attacking success (Smart et al., 2014; Tierney et al., 2017) that are indicative of success in RU (Hughes et al., 2017). However, differences in the importance of performance indicators are evident between the male and female games (Hughes et al., 2017). For example, a higher number of line breaks and ball carries correlate to winning in women's but not men's international rugby.

Game or training movement is measured through time‐motion analysis or GPS approaches. The predominant method for understanding the level of demand players perform is through application of thresholds describing pre‐selected speed zones (Bradley et al., 2020; Cahill et al., 2013; Cunniffe et al., 2009). Thresholds allow the categorisation of total distance into separate blocks or volumes, often associated with a qualitative descriptor such as walking, striding or sprinting and with quantification of the level of movement such as high‐speed or ‐intensity (Cunniffe et al., 2009; Suarez‐Arrones et al., 2014) that enables coaches to identify player activity, work intensity and volume. Absolute thresholds are useful and important within team sports as the game is played within the same absolute context and capacity in individual games. The ability to produce running speeds in direct comparison to the opposition faced will determine success or failure in a given challenge or match and players must produce physical performance relevant to the scenario occurring (Sweeting et al., 2017). As such, absolute thresholds, that treat all players as equal, are useful for convenience or when broadly comparing players within a team across a season, however inconsistency exists in the definition of thresholds across studies (Curtis et al., 2023).

The use of GPS‐based locomotor data in women's RU is gaining popularity. However, speed thresholds are usually based on arbitrary manufacturer guidance or data derived from the men's game (Bradley et al., 2020; Callanan et al., 2021; Nolan et al., 2023; Suarez‐Arrones et al., 2014; Woodhouse et el., 2021). For example, Suarez‐Arrones et al. (2014) and Bradley et al. (2020) applied the male‐specific thresholds from the work of Cunniffe et al. (2009) of 0–6 km^.^h^−1^ (walking), 6.1–12 km^.^h^−1^ (jogging), 12.1–14 km^.^h^−1^ (slow running), 14.1–18 km^.^h^−1^ (medium‐intensity running), 18.1–21 km^.^h^−1^ (high‐intensity running), >21.1 km^.^h^−1^ (sprinting). Evidence suggests the maximum speeds achieved by female players are 2–6 km.h^−1^ slower Suarez‐Arrones et al. (2014), while greater differences of 6–8 km.h^−1^ for players in the English Premier15 s (Bradley et al., 2020) compared to the English men's Premiership (Cahill et al., 2013). While the team‐wide in‐game mean maximum speed (20.5 km.h^−1^) for the forward players in the study by Bradley et al. (2020) was below the sprinting threshold implemented in the study based on the male game. In addition, the application of male‐derived speed thresholds to female performance is not limited to women's RU, with this reductive approach common across other football codes including rugby 7 s (Clarke et al., 2014), rugby league (Cummins et al., 2023), football (Gualtieri et al., 2023) and Australian rules football (Wing et al., 2021).

Due to the lower speeds attained by female players, male‐based thresholds may underestimate the true volume of HIR in women's rugby (Curtis et al., 2023). While an average decrease in total distance of 18.2% across female studies is observed (5291.8 ± 636.1 m) (Bradley et al., 2020; Callanan et al., 2021; Nolan et al., 2023; Sheppy et al., 2019; Suarez‐Arrones et al., 2014) compared to male data (6467.5 ± 381.6 m) (Cahill et al., 2013; Coughlan et al., 2011; Cunniffe et al., 2009), when utilising existing male‐derived thresholds, the distance at high‐intensity or sprinting speeds reported across female studies is 74.4% lower (183.5 ± 32.7 m) (Bradley et al., 2020; Callanan et al., 2021; Nolan et al., 2023; Suarez‐Arrones et al., 2014) than male players (717.3 ± 145.1 m) (Cahill et al., 2013; Cunniffe et al., 2009). This highlights the issue with utilising male‐based thresholds directly to women's rugby performance as the change in the proportion of high‐intensity locomotion is markedly greater than the change in total distance which might be expected if the thresholds were transferable.

While absolute thresholds are still commonplace due to their ease of application, it is appropriate that thresholds more specific to female players are developed and to address the gap in data available in female sport (Anderson et al., 2023; Cowley et al., 2021). In a recent systematic review, Curtis et al. (2023) recommended that further research into adjusted female‐specific thresholds during match demand analysis is warranted to address the potential underestimation of distances run at higher intensities due to the implementation of male‐derived thresholds. The aim of this study was to assess alternative GPS speed thresholds in comparison to a set of male‐derived thresholds, to identify the effect on movement distances and to make recommendations for use in women's 15‐a‐side RU.

## 2 MATERIALS AND METHODS

### 2.1 Participants and study design

A cross‐sectional study using time‐motion analysis was conducted on 58 Women's Premier15 s rugby players (30 Forwards, 28 Backs) across 18 league games. In total 178 individual player games were recorded for later analysis to determine changes in running distances with adjusted threshold values. Player characteristics (mean ± SD) were age 23.9 ± 4.5 years, height 1.71 ± 0.07 m, mass 74.5 ± 11.4 kg. All players were provided with a participant information sheet detailing the purpose of the study and the methods including the risks and benefits and a verbal explanation was provided in a subsequent team training meeting where players were given the opportunity to ask questions. Written informed consent was obtained from all players. The study was approved by the University of Sunderland [*Blinded*] Ethics sub‐group and followed the Code of Ethics of the World Medical Association (Declaration of Helsinki, 2013).

### 2.2 Maximal sprint testing

On grass field at the beginning of a training session after a 20‐min warm up, 47 of the 58 players performed two 20 m sprints, following the Rugby Football Union testing guidelines for Premier15 s teams. While we appreciate that maximal running speed is usually achieved at distances of 30–50m, shorter distances are appropriate to elicit maximum speeds as 96% of V_MAX_ achieved at 21 m in RU (Barr et al., 2013) and 23.1 m in sprinters (Nagahara et al., 2018), while the majority of sprint distances in rugby league are <20 m (Gabbett, 2012). Photoelectric timing gates (Smartspeed, Fusion Sport Inc., Reading UK) were set up 2 m apart at 0 and 20 m distances, with an additional dummy pair of timing gates positioned at 25 m. Players were instructed to sprint as quickly as possible through to the gates at 25 m. The dummy gates were included to reduce the likelihood of players slowing down before reaching the gates at 20 m to maximise velocity at this point. Players received a five‐minute passive rest between the two sprints. Time taken to cover the 20 m distance was recorded and maximum velocity (V_MAX_) was calculated by dividing the distance by the time. The higher of the two velocities was taken as the players' actual V_MAX_ and the mean value for the whole team was calculated from individual player V_MAX_ to determine the adjusted speed thresholds. It should be noted that the mean V_MAX_ recorded from the linear sprints (21.4 km.h^−1^) was higher than the mean in‐game recorded speed from the GPS devices (20.7 km.h^−1^).

### 2.3 Match analysis measurement

Movement data was captured on all players using 10 Hz GPS units (Catapult Minimax S4 GPS, Catapult Innovations, Melbourne, Australia) in a harness, positioned between the shoulder blades following manufacturer's guidelines. Catapult 10 Hz GPS devices have been shown to have acceptable levels of validity compared to similar devices (Cummins et al., 2013; Johnston et al., 2014) and display low levels of measurement error (Rampinini et al., 2014), though it has been reported that the likelihood of error in measuring high‐intensity distances increases at speeds ≥20 km.h^−1^ (Johnston et al., 2015). GPS units were switched on at least 10 min prior to the start of each game to acquire a satellite signal with the horizontal dilution of precision values between 0.64 and 1.76 across all matches. Data were trimmed to include only playing time, so GPS data only described on‐field player activity but included any play stoppages such as scrum resets or injuries. All data were downloaded using the Catapult Sprint 5.03 software (Catapult Innovations, Melbourne, Australia) with specific speed thresholds applied to calculate player movement data.

### 2.4 Speed threshold adjustments

To identify the effect of female‐specific speed thresholds on the match characteristics of player, three sets of thresholds were applied to each data file (Table 1). Rugby‐standard absolute thresholds, derived from male players (Cunniffe et al., 2009), were applied and taken as the reference to enable comparison: Abs_Male_ 0–6 km^.^h^−1^ (walking), 6.1–12 km^.^h^−1^ (jogging), 12.1–14 km^.^h^−1^ (slow running), 14.1–18 km^.^h^−1^ (medium‐intensity running), 18.1–21 km^.^h^−1^ (high‐intensity running), ≥21.1 km^.^h^−1^ (sprinting). Reduced‐speed thresholds (Abs_FVmax_) were applied, with sprints classified as 90% *V*_max_ (Reardon et al., 2017) of the team attained during the linear sprint tests (21.4 km/h) and the speed required to elicit high‐speed running relative to the second ventilatory threshold (VT_2speed_) (Buchheit et al., 2009; Scott et al., 2018) derived from the 30–15 intermittent fitness test, where VT_2speed_ was 87% of sprinting velocity. The final thresholds (Abs_Female_) were set based on the work by Clarke et al. (2014) who suggested that a speed of 3.6 m·s^−1^ (12.6 km·h^−1^) is more indicative of high‐speed running than 5 m·s^−1^ (18 km·h^−1^) in women's rugby sevens. This equates to 72% of the male‐derived speed thresholds and the Abs_Male_ thresholds were reduced on a ratio of 0.72.

**TABLE 1:** Speed threshold zones (all values in km^.^h^−1^ with corresponding values in [m·s^−1^]).

Threshold	Walking	Jogging	Slow running	Medium‐intensity running	High‐intensity running	Sprinting
Abs_Male_	0–6.00 [0–1.67]	6.10–12.00 [1.68–3.33]	12.10–14.00 [3.36–3.89]	14.10–18.00 [3.92–5.00]	18.10–21.00 [5.03–5.83]	≥21.10 [≥5.86]
Abs_FVmax_	0–5.00 [0–1.39]	5.10–9.90 [1.42–2.75]	9.91–12.85 [2.76–3.56]	12.86–16.70 [3.57–4.63]	16.71–19.25 [4.64–5.34]	≥19.26 [≥5.35]
Abs_Female_	0–4.32 [0–1.20]	4.33–8.64 [1.21–2.40]	8.65–10.08 [2.41–2.80]	10.09–12.96 [2.81–3.60]	12.97–15.12 [3.61–4.20]	≥15.13 [≥4.21]

### 2.5 Data analysis

All data were exported from Catapult Sprint to Microsoft Excel (Microsoft Co., Redmond, WA) for further analysis. Descriptive movement data (mean ± SD) for each speed threshold set were calculated and percentage differences between total distance (*m*) in HIR and sprint (Spr) zones were calculated. Data were analysed using a one‐way ANOVA in SPSS v25 (IBM Co., Armonk, NY) to determine significant differences in total and speed zone distances between the thresholds. Effect sizes (Cohen's d) and 95% confidence intervals were calculated to identify the magnitude of observed changes and forest plots were created to visualise the difference in effect size relative to the reference data (Abs_Male_). The magnitude of the effect sizes were classified as trivial (0–<0.2), small (≥0.2–<0.6), moderate (≥0.6–<1.2), large (≥1.2–<2.0) and very large (≥2.0–<4.0) (Hopkins et al., 2009).

## 3 RESULTS

**FIGURE 1:**
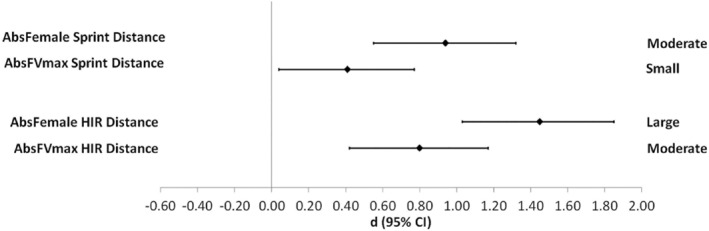
Forest plot indicating the differences between high‐speed running and sprint distances due to the adjusted absolute speed thresholds from Abs_Male_ (*d* = 0). Centre mark indicates the effect size and bars indicate 95% confidence intervals.

HIR and sprinting (Spr) distances using the male‐derived thresholds (Abs_Male_) were 63 ± 71 m and 30 ± 53 m respectively (Table 2). Significantly greater distances at higher intensity speeds were observed when female‐adjusted thresholds were applied, with a moderate increase in HIR distance of 231% (139 ± 115 m, *p* = 0.01, ES 0.80) and a small increase in Spr distance of 200% (60 ± 90 m, *p* = 0.131, ES 0.41) with the Abs_FVmax_ thresholds (Figure 1). Similarly, a large increase in HIR distance of 379% (239 ± 157 m, *p* < 0.01, ES 1.45) and a moderate increase in Spr distance of 456% (137 ± 152 m, *p* < 0.01, ES 0.94) with the Abs_Female_ thresholds were observed. Of the 58 players who participated in the study, only 24 (41%) achieved speeds greater than the 21 km·h^−1^ sprint threshold from the male‐derived thresholds. At the Abs_FVmax_ threshold this increased to 44 (76%), with all players at the Abs_Female_ threshold reaching speeds above the sprint threshold.

**TABLE 2:** Changes in high intensity running and sprinting variables across the three speed thresholds.

Threshold	HIR distance (m)	Sprint distance (m)
Abs_Male_	62.5 ± 71.3	29.7 ± 52.5
Abs_FVmax_	139.4 ± 115.5^a^	59.7 ± 90.4
Abs_Female_	238.9 ± 156.8^a^	137.1 ± 151.9^a^

^a^ Indicates a significant increase compared to Abs_Male_ (*p* < 0.05).

## 4 DISCUSSION

The aim of this study was to assess two alternative female‐related GPS speed thresholds in comparison to a set of male‐derived thresholds, to identify the effect on high‐intensity movement distances and to make recommendations for use in women's 15‐a‐side RU. Adjustments to the speed thresholds resulted in significantly small to moderate increases in total sprint distances and significantly moderate to large increases in total high‐intensity running distances completed by a group of female rugby players.

Total high‐intensity locomotion calculated using male‐derived thresholds (92.3 ± 123.8 m) equated to 2.1% of the total distance, which is similar to the proportions of between 2.7% and 4.9% previously reported in women's RU (Bradley et al., 2020; Callanan et al., 2021; Nolan et al., 2023; Suarez‐Arrones et al., 2014), indicating an underestimation of total high‐intensity locomotion in both the current and previous studies. After adjusting the speed thresholds to reflect the lower maximum speed achieved by female players (Abs_Female_), high intensity locomotion increased to 8.2% and aligns more closely to that observed during men's English Premiership match‐play of between 7.6% and 10.6% (Coughlan et al., 2011; Cunniffe et al., 2009) but still below the 14.3% reported by Cahill et al. (2013). This likely overestimated high‐speed running value is probably due to Cahill and colleagues' classification of higher intensity running as >50% maximum velocity under the term ‘striding’ rather than defining moderate‐ and high‐intensity running as found in the current and other studies. The adjustment magnitude of 0.72 of male velocity thresholds applied in the current study was based on the work of Clarke et al., 2014. This is in line with an in‐game mean maximum velocity (20.7 km^.^h^−1^) in the current study being 73% of men's RU in‐game mean maximum velocity of 28.5 ± 2.4 km^.^h^−1^ (Cahill et al., 2013; Coughlan et al., 2011; Cunniffe et al., 2009), indicating that an adjustment magnitude of 0.72 for the Abs_Female_ thresholds is an appropriate value to equitably reflect the differences in high intensity activity in the women's game. This can be related to previous findings that maximum speeds achieved by female players are 2–8 km.h^−1^ slower (Bradley et al., 2020; Suarez‐Arrones et al., 2014), so female players are less likely to achieve the male‐derived threshold speeds that demarcate high‐intensity running and sprints. This is supported in the current study, as only 41% of female players were able to achieve maximum speeds of greater than 21 km·h^−1^ so more than half of all players would not cover any distance that would be classed as a sprint when male thresholds were implemented.

It has previously been proposed that running speed at the second ventilatory threshold (VT_2speed_) is indicative of HIR (Buchheit et al., 2009; Scott et al., 2018) and adjusting GPS speed thresholds relative to the VT_2speed_ provides a physiologically supported value rather than an arbitrary % of maximum velocity. Such an approach was implemented in international women's rugby 7 s (Clarke et al., 2014), where the application of a VT_2speed_ threshold increase the proportion of high‐intensity running from 14% to 37% compared to the male threshold of 5 m·s^−1^. However, in the current study, the VT_2speed_ based Abs_FVmax_ thresholds only resulted in an increase in the proportion of high‐intensity activity to 4.5% of total distance, possibly due to the higher absolute HIR speed of 16.7 km^.^h^−1^ the VT_2speed_ adjustment of 87% V_Max_ applied compared to the 12.6 km^.^h^−1^ threshold (Clarke et al., 2014). This may be due in part to the application of the 87% V_Max_ value proposed by Buchheit (2009) in male sprinters/footballer players and that VT_2speed_ thresholds may not reflect a similar boundary for high‐intensity activity in female 15‐a‐side RU players. However, no similar %V_Max_ at ventilatory thresholds is available in female athletes that could be utilised in the current study. It is likely that the %V_Max_ that the female players attained VT_2speed_ threshold is lower than 87% V_Max_ boundary and is likely to have resulted in part to the continued underestimation of high‐intensity activity (Clarke et al., 2014). To address this issue, it would be prudent to perform individual ventilatory threshold assessments of the team in the future to identify the actual VT_2speed_ threshold or to determine the validity of the 87% V_Max_ value in 15‐a‐side RU. This may facilitate individualisation of speed thresholds as an unintended outcome. Additionally, tactical and technical differences exist between rugby 7 s and 15‐a‐side versions of the sport and greater relative volumes of running are expected in 7 s rugby. While the use of VT_2speed_ calculated thresholds has a physiological rationale, adopting these thresholds continues to be underestimated in women's 15‐a‐side RU.

To our knowledge, three further studies in addition to Clarke et al. (2014), have utilised adjusted speed thresholds in women's RU. Also in rugby 7 s, Misseldine et al. (2021) reported an increase in sprint distances for the forwards (60 ± 32 m vs. 171 ± 45 m) and backs (112 ± 69 m vs. 216 ± 88 m), representing a % total distance increase from 4%–8% to 11%–14%, with a female adjusted sprint threshold of 61% of female player's maximal speed that is reflective of the male sprint threshold of 5.6 m/s that is 61% of male V_MAX_. Similar to the findings in the Clarke et al. (2014) paper, the greater sprint distances in these studies are likely due to the differences in tactical play in 7 s rugby and potentially higher maximal in‐game velocities, however, a direct comparison is limited as the adjustment was specific to sprint events and did not examine the impact on running distances. In an examination of worst‐case scenario (WCS) locomotor demands in women's international 15‐a‐side RU, Sheppy et al. (2019), a threshold of >4.4 m·s^−1^ was used to calculate high‐speed running distances, which was approximately 60% of the mean maximal velocity of the squad and a value that is 88% that of the arbitrary male threshold of 5 m·s^−1^. While the application of such a threshold makes the calculation of WCS more relevant for the women's game, the authors did not directly compare against male thresholds and the lack of detail on the game duration make comparison to the findings in the current study limited. Our findings are consistent with those of Busbridge et al. (2022), who reported mean high‐speed running distances of 452 ± 241 m performed at speeds >16.7 km^.^h^−1^, compared to 376 ± 154 m and a similar proportion of total distance (7.9%). While the high‐speed threshold is the same in both studies, Busbridge and colleagues selected the thresholds based on research on female soccer and hockey which have different physical demands than RU.

### 4.1 Limitations

The study aimed to examine the effect of adjusted speed thresholds on high‐speed locomotor distances. We have created two versions to correct for the underestimation of these distances when using male‐thresholds. While we have attempted to support and justify the magnitude of change relative to the VT_2speed_ and % difference in maximum velocity to produce the new thresholds. However, a consensus on the definition of high‐speed running and sprinting would aid in the identification of thresholds (Sweeting et al., 2017) and alternative values may be more appropriate, such as the ∼60% of maximum velocity have been applied to female 15‐a‐side (Sheppy et al., 2019) and 7 s rugby (Misseldine et al., 2021) and these lower high‐speed thresholds are likely to produce further increases in distances at high intensities to bring them in line with male values, such as Cahill et al. (2013). Alternative approaches to identifying specific thresholds, for example, statistical processes such as spectral clustering used by Park et al. (2019) in women's football, could be investigated to reduce the arbitrary nature of the threshold values. When applied to female rugby league data (Cummins et al., 2023), the same spectral clustering techniques were successful in identifying practical velocity thresholds (0–11.49 km/h: low velocity; 11.50–17.49 km/h: moderate velocity; 17.50–20.99 km/h: high velocity; and >21.00 km/h: very‐high velocity). While these high‐intensity thresholds are greater than proposed in the current study, the tactical and technical demands differ between the two rugby codes that are likely to account for the variation in thresholds, however the work of Cummins and colleagues does show the potential in application to female RU data. Furthermore, the adjusted thresholds still produce absolute thresholds that are applied consistently across the whole team. This has been reported to both under‐ and over‐estimate distance dependent on each player's specific maximum velocity (Gabbett, 2015) and individualised thresholds are suggested to account for such differences by normalising the distances. A benefit of using absolute values is the ease of application in the GPS data processing phase, with individualised thresholds requiring a greater technical knowledge and resource requirement that may delay the processing and delivery of player metrics. Additionally, a decision is needed on the source of the maximum velocity to calculate thresholds (Sweeting et al., 2017), usually either obtained from sprint testing or in‐game speeds, such as the in‐game sprints that were classified as velocities greater than 90% *V*_max_ (Reardon et al., 2017). Future research could examine the development of female‐related individualised thresholds. Finally, we have only calculated the change in high‐speed distance during matches and the impact of female‐specific distances on training loads, match preparation or performance outcomes would support the wider use of female‐specific data in the women's game.

## 5 CONCLUSION

Where absolute speed thresholds are used to interpret movement patterns in women's 15‐a‐side RU, it is prudent to use female‐specific values as these will more appropriately represent the maximal speeds attained by female players. We suggest that reducing the speed thresholds to those presented in this paper, specifically the Abs_Female_ as these produce high‐intensity speed distances that are proportional to male rugby players. This will produce locomotor data that is relevant to actual demands experienced in the women's game, especially for high‐intensity activity that is likely to have a greater impact on player performance and game outcomes. Therefore, this will allow coaches to prepare players more adequately through training at female‐specific intensities.

## CONFLICT OF INTEREST STATEMENT

The authors report there are no competing interests to declare and no funding was received for the completion of this work.

## Data Availability

The participants of this study did not give written consent for their data to be shared publicly, so due to the sensitive nature of the research supporting data is not available.
